# Infections on Temporal Networks—A Matrix-Based Approach

**DOI:** 10.1371/journal.pone.0151209

**Published:** 2016-04-01

**Authors:** Andreas Koher, Hartmut H. K. Lentz, Philipp Hövel, Igor M. Sokolov

**Affiliations:** 1 Institut für Theoretische Physik, Technische Universität Berlin, Hardenbergstraße 36, 10623 Berlin, Germany; 2 Institute of Epidemiology, Friedrich-Loeffler-Institute, Südufer 10, 17493 Greifswald, Germany; 3 Bernstein Center for Computational Neuroscience Berlin, Humboldt Universität zu Berlin, Philippstraße 13, 10115 Berlin, Germany; 4 Institut für Physik, Humboldt-Universität zu Berlin, Newtonstraße 15, 12489 Berlin, Germany; Universidad Rey Juan Carlos, SPAIN

## Abstract

We extend the concept of accessibility in temporal networks to model infections with a finite infectious period such as the susceptible-infected-recovered (SIR) model. This approach is entirely based on elementary matrix operations and unifies the disease and network dynamics within one algebraic framework. We demonstrate the potential of this formalism for three examples of networks with high temporal resolution: networks of social contacts, sexual contacts, and livestock-trade. Our investigations provide a new methodological framework that can be used, for instance, to estimate the epidemic threshold, a quantity that determines disease parameters, for which a large-scale outbreak can be expected.

## Introduction

Networks are one of the most important ways to represent a finite set of elements with complex interaction patterns. As vast amount of data becomes publicly available, the analysis of complex networks plays an ever increasing role throughout different areas such as computer science, physics, social science and biology. Well known applications are the analysis of the World Wide Web [1], scientific collaborations [2] and protein interaction networks [3] to name only a few.

It has become important to not only analyse network structures using graph theoretical tools, but to conduct numerical experiments [4, 5]. This approach has led to numerous advances in epidemiological modelling, because it allowed to analyse the impact of the topology on the dynamics of infections [6, 7]. It was a major conceptual improvement to consider heterogeneous contact patterns in face of previous models, which assumed homogeneous mixing among the individuals and separated the population into compartments such as susceptible (S), exposed (E), infected (I) and recovered (R) [8]. As a result new vaccination strategies have been suggested [9, 10] and network-based models are promising candidates to forecast and mitigate the impact of global epidemics [5, 11] in the future.

**Fig 1:**
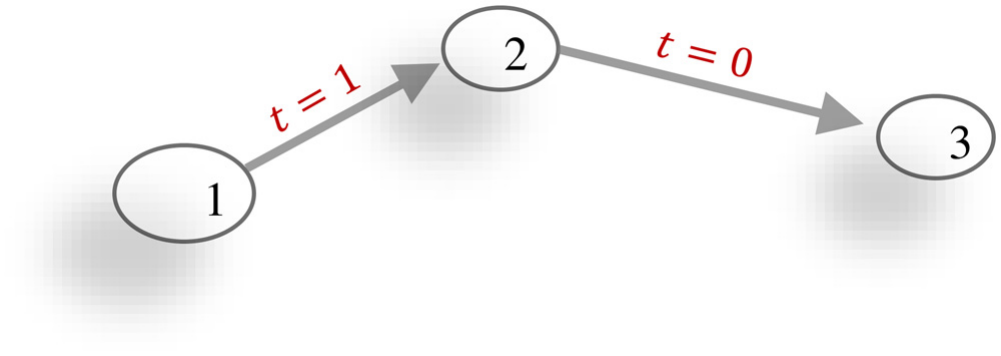
Transitivity is not assured in temporal networks. Here, links 1 → 2 and 2 → 3 exist, but the temporal order (1 → 2 at time *t* = 1 and 2 → 3 at time *t* = 0) prevents information to spread from node 1 to 3.

However, methods from classic network analysis do not take into account the temporal resolution of contact data. In particular, a major drawback arises from the fact that the number of paths relevant for the disease spreading can be strongly overestimated [12, 13]. This is a consequence of a factitious transitivity, if the temporal information of the links is neglected (see Fig 1).

The availability of data with high temporal resolution [14–17], so called *temporal networks*[18, 19], offers a promising way to improve epidemiological models. This approach allows to consider only infection paths, with links that appear in the correct temporal order—a main conceptual and practical advantage. Taking into account these time-respecting paths overcomes the limitation inherent to the static approach for realistic investigations of disease transmission.

A natural way to include the causal sequence of edges into the analysis is to consider accessibility graphs. Here, a directed edge between two nodes appears only if one is accessible by the other through a time-respecting path. In other words, an accessibility graph provides information whether a temporal path connects two nodes within a certain time period. Recently, an analytical approach has been proposed to calculate the corresponding accessibility matrix, which was termed *unfolding accessibility* [13, 20]. This mathematical description is based on a list of adjacency matrices, whose elements refer to the snapshots of the temporal network under consideration. The approach models a susceptible-infected type of disease, i.e. an infection without recovery. Here, each contact leads to newly infected neighbours in a deterministic way, thus, serving as a worst-case scenario.

In this paper, we generalize the concept of accessibility and introduce a fixed recovery time. This allows us to include diseases with a finite infection period, and we will consider the widely used susceptible-infected-recovered (SIR) model. As in the previous work [13], we base our formalism on elementary matrix operations with Boolean arithmetic, which allows us to integrate the disease and network dynamics in an unified framework.

From the modelling perspective our formalism gives us the advantage that we take into account the full temporal and topological complexity of the interaction patterns, without restricting ourselves to limiting assumptions such as Markovian dynamics [21], pair approximations [22], individual-based [23] and degree-based mean-field theory [6]. This is a consequence of our approach to use time-ordered products of exact adjacency matrices, which preserve the causal appearances of links. Furthermore, the concept of accessibility matrices allows us to track the individual infection status. Thus, no dynamic approximation on the number of contacts between susceptible and infected, which are crucial for disease transmission, is needed.

The remaining three sections of the paper are structured as follows. First, we will introduce the terminology of the state vector formalism, which we will then generalize to a matrix formalism. To demonstrate the power of our approach, we will analyse disease dynamics on different empirical networks: networks of social contacts, sexual contacts, and livestock-trade. This will allow us, for instance, to estimate the critical infectious period. Finally, we will summarize our results.

## Materials and Methods

### Epidemiological Model: State Vector Formalism

Since the beginning of modern epidemiological modelling [24] a Markovian dynamics was typically assumed. Upon a contact between an infected and a susceptible individual [*SI*] the disease can be transmitted with a probability *α*. Moreover, infected individuals [*I*] recover at each time step with a probability *β*:

$$\begin{array}{cc} {\left[S\right]}_{t+1} & =1-{\left[I\right]}_{t}-{\left[R\right]}_{t} \end{array}$$ (1a)

$$\begin{array}{cc} {\left[I\right]}_{t+1} & ={\left[I\right]}_{t}+\alpha {\left[SI\right]}_{t}-\beta {\left[I\right]}_{t} \end{array}$$ (1b)

$$\begin{array}{cc} {\left[R\right]}_{t+1} & ={\left[R\right]}_{t}+\beta {\left[I\right]}_{t}. \end{array}$$ (1c)

Starting from this set of equations, a number of approaches have been proposed to approximate the interaction term [*SI*], which includes all topological features of the underlying contact structure [25]. In this paper we will identify the critical contacts [*SI*] directly from the temporal network. Eqs (1a) to (1c), which describe the widely used SIR model, serve as a reference before elaborating on our algebraic alternative.

We start from a system with a fixed number of nodes *N*. A node can be either an individual or a separate population, which is however treated as a unit in the sense that it enters one epidemiological state: susceptible (S), infectious (I) or recovered (R). A contact between two nodes appears as a link in the network at the given time and can be either undirected, which is naturally the case for proximity data [14, 26] or, directed if trade is considered for example [27].

The interactions in a static network are typically represented by an adjacency matrix *A* with a positive entry *A*_*ij*_ = 1 if node *j* is connected to *i* and *A*_*ij*_ = 0 otherwise. In this paper we will allow for directed links and therefore we will assume asymmetric adjacency matrices in general.

A temporal network can be defined in a variety of ways, depending on the underlying contact data and its purpose [19]. We adapt a widely used approach, which assumes that the temporal network evolves in equidistant time steps, given by the sampling resolution of the data. It allows us to define the temporal network conveniently by a set of adjacency matrices {*A*_*t*_}. Here, each element *A*_*t*_ describes a snapshot of interactions at time *t* = 1, …, *T*, with *T* being the observation time. This is a valid approximation as long as the time to form a link, e.g. the latency in case of transport connections, can be neglected compared to the sampling time [19]. Furthermore, we will use the highest resolution available throughout the paper in order to minimize potential errors. This happens due to the fact, that causal appearances of links can be violated if snapshots are partially aggregated. As a result, potential outbreaks may be systematically overestimated [13].

If we assume that the pathogen can only be passed to the nearest neighbours within every snapshot, then a temporal network allows us to infer the critical contacts [*SI*] (Eq (1b)) directly from the disease status of every node. The assumption can be justified if the temporal resolution prohibits consecutive interactions within one time step and is an approximation in the case of partially aggregated data. Therefore, we can use the time-depending topology in order to determine the disease dynamics directly.

We begin by replacing the fraction of infected [*I*] and recovered [*R*] nodes in Eq (1b) by Boolean state vectors:

$$\begin{array}{cc} \left[I\right] & \to {\left(i\right)}_{k}=\left\{\begin{array}{cc} 1, & \text{node}\;k\;\text{is}\;\text{infected}\\ 0, & \text{otherwise} \end{array}\right. \end{array}$$ (2a)

$$\begin{array}{cc} \left[R\right] & \to {\left(r\right)}_{k}=\left\{\begin{array}{cc} 1, & \text{node}\;k\;\text{is}\;\text{recovered}\\ 0, & \text{otherwise.} \end{array}\right. \end{array}$$ (2b)

Following the binary nature of the state vectors, we will only use Boolean arithmetic [28] without explicitly indicating it. Hence, element-wise addition and scalar multiplication are replaced by the logical “or” and “and”, respectively. Matrix multiplication is handled similarly through the definition (*A* ⋅ *B*)_*ij*_ = ∑_*k*_ *A*_*ik*_ *B*_*kj*_ and element-wise multiplication ∘ is defined by (*A* ∘ *B*)_*ij*_ = *A*_*ij*_ *B*_*ij*_. Finally, we introduce the element-wise negation ¬ of a vector or a matrix, i.e. (¬*A*)_*ij*_ = 0 if *A*_*ij*_ = 1 and (¬*A*)_*ij*_ = 1 otherwise.

Furthermore, we will consider the worst-case scenario, where the disease is transmitted upon contact, i.e. *α* = 1 in Eq (1b). It is a convenient way to separate topological and probabilistic fluctuations in a temporal network and to concentrate on the first. This assumption, is not crucial and we will sketch now one possible generalization to an arbitrary *α* ≤ 1.

If an infected node does not transmit the disease to its susceptible neighbour with a probability 1−*α*, we can just as well neglect the link with the same probability. This idea can be formalized by a stochastic operator Ω(*A*), which acts element-wise on the adjacency matrix *A*: For *A*_*ij*_ = 0 we define Ω(*A*_*ij*_) = 0 and for positive entries *A*_*ij*_ = 1 we choose Ω(*A*_*ij*_) = 1 with probability *α* ≤ 1 and Ω(*A*_*ij*_) = 0 otherwise. The original set of adjacency matrices *A*_*t*_ (for *t* = 1, …, *T*) would have to be replaced by the thinned out modification ${\tilde{A}}_{t}=\Omega \left(A_{t}\right)$, in order to obtain one realization. Finally, the ensemble of all possible outbreak scenarios would allow us to calculate average values. This approach inherits from the simplified model with *α* = 1, the advantage to account for dynamic and topological correlations. It increases, however, the computational effort and mixes topological with probabilistic effects. In order to focus on the former we will therefore exclude stochastic fluctuations and choose *α* = 1.

Using the explicit contact structure given by the set of static snapshots {*A*_*t*_}, we propose the following model for the SIR dynamics:

$$\begin{array}{cc} i_{t+1} & =\left(A_{t}i_{t}+i_{t}\right)\circ \left(\neg \;r_{t+1}\right) \end{array}$$ (3a)

$$\begin{array}{cc} r_{t+1} & =\sum_{k=0}^{t+1-\tau }i_{k}. \end{array}$$ (3b)

The number of newly infected individuals [*SI*] is given by the product *A*_*t*_ *i*_*t*_ in Eq (3a), where *A*_*t*_ is the *N* × *N* dimensional adjacency matrix of the temporal network at time *t* = 1, 2, …, *T*. A node enters the recovered state *I* → *R* (Eq (3b)) if it has been infectious for *τ* time steps. Therefore this model, contrary to Eqs (1a)–(1c), is not Markovian, i.e. the state of the system depends not only on the previous, but on the last *τ* time steps. This is a frequently used simplification [29, 30], which leads to a very efficient implementation. Finally, the element-wise operation ∘(¬*r*_*t*+1_) guarantees that a recovered node cannot be reinfected or pass a disease.

The system of Eq (3) describes the disease dynamics, which starts with arbitrary initial conditions *i*_0_ and *r*_0_. From a conceptual and practical point of view, it is advisable to generalize the state vector to a matrix formalism that accounts for different initial conditions at the same time. This will be subject of the next section.

### Tracking All Initial Conditions: Matrix Formalism

It is often important to evaluate the role of single nodes in a contact network with respect to their influence on the outbreak dynamics. For example, one could look for nodes with a high temporal out-component (virulence) [12] or nodes with a high probability of catching a disease (vulnerability) [31]. This consideration motivates us to choose the initial conditions as *i*_0_ = *e*_*k*_ and *r*_0_ = **0** indicating that only the node *k* is infected and all other nodes are susceptible. *e*_*k*_ denotes the *k*-th column of the identity matrix, where (*e*_*k*_)_*i*_ = 1, if *k* = *i* and 0 otherwise. Instead of repeatedly considering Eq (3) for every *e*_*k*_, *k* = 1, …, *N*, it will prove useful to stack all initial vectors into *N* × *N* matrices:

$$\begin{array}{cc} I_{t=0} & =\left[e_{1},e_{2},\cdots ,e_{N}\right]=1_{N} \end{array}$$ (4a)

$$\begin{array}{cc} R_{t=0} & =\left[0,0,\cdots ,0\right]=0_{N}. \end{array}$$ (4b)

This approach allows us to convert the state-vector description into a matrix-based formalism, which enables us to consider all homogeneously sampled, initial conditions simultaneously:

$$\begin{array}{cc} I_{t+1} & =\left(A_{t}I_{t}+I_{t}\right)\circ \left(\neg \;R_{t+1}\right) \end{array}$$ (5a)

$$\begin{array}{cc} R_{t+1} & =\sum_{k=0}^{t+1-\tau }I_{k}, \end{array}$$ (5b)

The *k*th column of the matrix *I*_*t*_ indicates the nodes that are infected at time *t* if the epidemic starts from the initial node *k*. As we are working with Boolean arithmetic, we only have binary entries in *I*_*t*_. Therefore, we can regard it as an adjacency matrix of an accessibility graph: A directed edge appears only if a time-respecting path exists that connects the initial node with the target and satisfies the epidemiological constraints: An infection can only be passed on within a fixed time window of size *τ* and a node cannot be reinfected.

In the special case, if the infectious period *τ* exceeds the observation time *T*, we are effectively dealing with an SI-type of infection. Then, we do not account for recovered nodes and thus all operations including the matrix *R* can be ignored. Note that the simplified expression of Eq (5a) *I*_*t*+1_ = *A*_*t*_ *I*_*t*_+*I*_*t*_ can be solved directly

$$\begin{array}{c} I_{t+1}=\prod_{k=0}^{t}\left(A_{k}+1\right), \end{array}$$ (6)

which recovers a result that was independently presented in [13] and [20]. The formalism described by Eq (6) was termed *unfolding accessibility* as the corresponding accessibility graph accumulates edges, which point from the initial seed to all nodes accessible via a time-respecting path within the given time frame. From a graph-theoretical point of view, adding a unity matrix to an adjacency matrix, i.e. $A_{k}+1$ in Eq (6), introduces a self loop to every node. It means that a path from a node back to itself is always possible. This circumstance mimics an infinite recovery time for disease propagation since a node can always be the source of a new transmission path. The unfolding accessibility itself is a special case of an earlier work [20], which is a temporal generalization of the Katz centrality [32] and therefore accounts for walks that visit a node repeatedly.

The matrix *I*_*t*_ of Eq (5a) provides a direct mapping from the seed of infection to the infected individuals. Therefore, we will also refer to it as the prevalence matrix. Given *I*_*t*_, we can derive the incidence matrix *J*_*t*_, which links the source nodes to the newly infected ones. Similarly, we introduce the cumulative incidence matrix *C*_*t*_, which maps the seed of infection to all nodes that have been infected up to the observation time *t*.

$$\begin{array}{cc} J_{t+1} & =I_{t+1}\circ \neg I_{t} \end{array}$$ (7a)

$$\begin{array}{cc} C_{t+1} & =\sum_{k=0}^{t+1}I_{k}. \end{array}$$ (7b)

In order to visualize the results we will use the density *ρ* of a matrix. It is defined by the ratio between the number of non-zero elements and the squared size of the matrix *N*^2^. Note that the three scalar values *ρ*(*I*_*t*_), *ρ*(*J*_*t*_) and *ρ*(*C*_*t*_) reflect the mean prevalence, incidence and cumulative incidence, respectively. That is, we automatically average over the ensemble of all homogeneously sampled initial conditions or in other words, we implicitly consider every node as a seed of infection. Similarly, we define *ρ*_*k*_ to be the ratio between the number of non-zero elements in the *k*-th column and the network size *N*. Thus, *ρ*_*k*_ is the fraction of infected individuals for one particular source node.

From the topology of the three accessibility graphs, which we have defined in this section, we can directly analyse the dynamics of a disease. To demonstrate the feasibility of the developed matrix formalism, we will apply our approach in the next section to three real-world networks.

## Application to Empirical Contact Networks

### Social Contacts Network

As a first example, we consider a social contact graph [26] that has been recorded during a three-days conference. Each of the 113 participants corresponds to a node and as soon as a proximity sensor detected a face-to-face contact over a period of 20 s an edge was generated. As a result one obtains a sequence of adjacency matrices, which refers to the snapshots of the interaction dynamics with a temporal resolution of 20 s. The data on social contacts allows to analyse the spread of airborne diseases as well as the propagation of information. With our model, we will hereby focus on the number of potential transmission paths.

**Fig 2:**
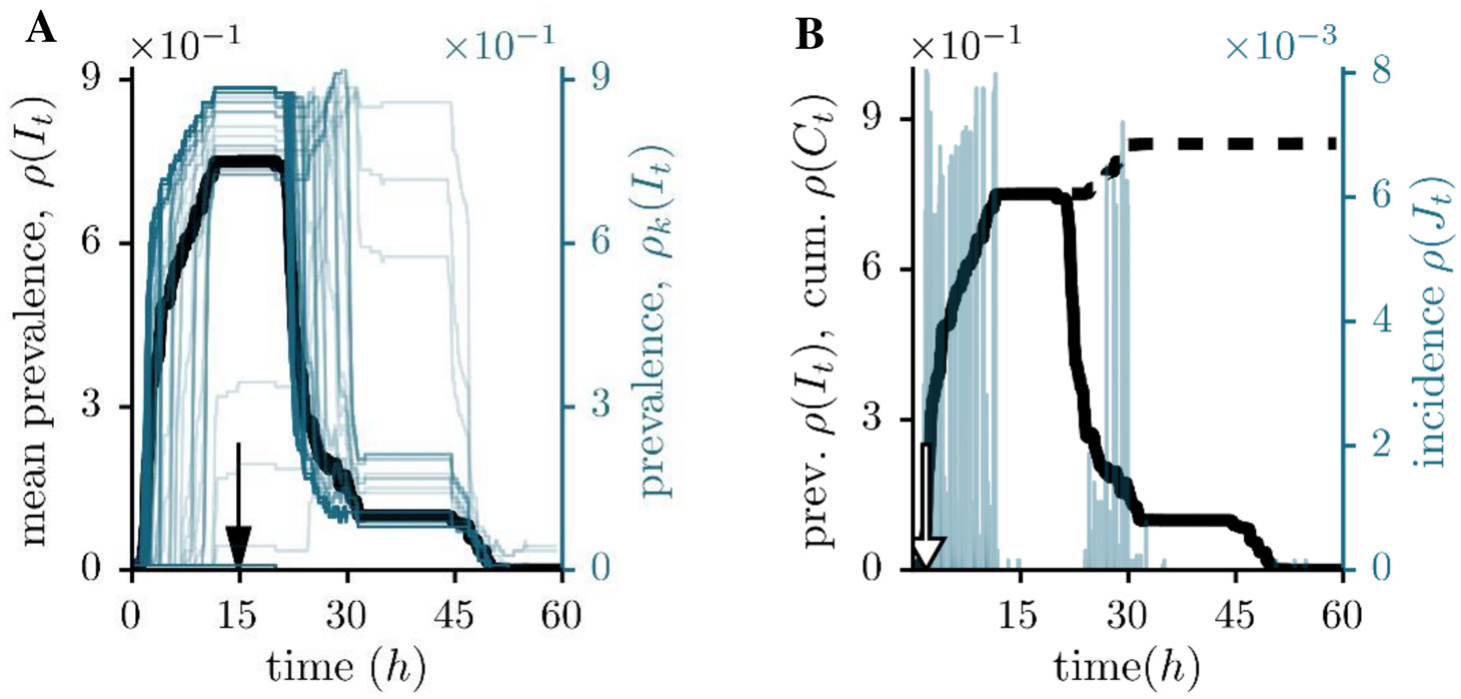
Prevalence, incidence and cumulative incidence for the social contacts network. (A) Comparison between the individual single source outbreaks (blue, right axes) and the corresponding, averaged prevalence (black, left axes). The infectious period is fixed at *τ* = 20h. The arrow at *t* = 14.8h indicates the maximum averaged prevalence. (B) Mean prevalence *ρ*(*I*_*t*_) (solid curves), incidence *ρ*(*J*_*t*_) (blue bars, right scale) and cumulative incidence *ρ*(*C*_*t*_) (dashed curves). Here, the arrow points at the maximum averaged incidence (*t* = 1.8h).

Fig 2 depicts an SIR process on the social contacts graph with a fixed recovery time of 20 hours. This choice is motivated by the characteristics of the dataset, i.e. a night break of several hours. Starting from one seed of infection, we observe a quick rise in prevalence *ρ*(*I*_*t*_) (Fig 2A) within only a few hours. This observation can be explained by a high number of changing interactions, which is the purpose of conferences. After around 21 hours the prevalence drops just as quickly, whereas one can observe two rather stable phases in between. These are reflected by plateaus in the cumulative incidence *ρ*(*C*_*t*_) (Fig 2B, dashed curve). They correspond to periods of vanishing incidence *ρ*(*J*_*t*_) (Fig 2B, blue bars) and are due to the fact that no interactions have been recorded during the night. The spreading process disappears before the third day of the conference. Despite the short infection time, about 85% of all nodes will be affected on average (Table 1).

**Table 1:** Comparison between the temporal networks and the time aggregated networks.

	$\rho (C_{T}^{\text{temporal}})$	$\rho (C_{T}^{\text{static}})$
social interactions	0.85	1.0
sexual contacts	0.027	0.88
livestock-trade	0.0048	0.15

**Fig 3:**
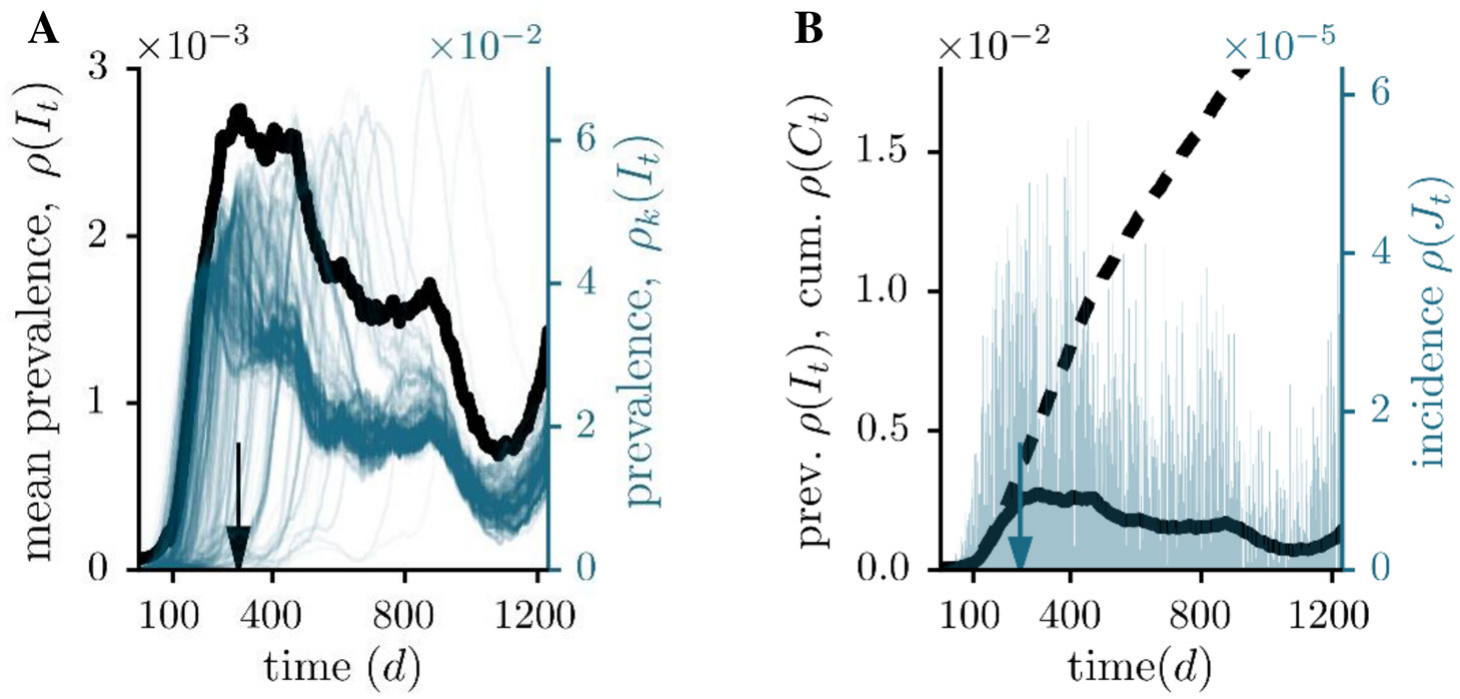
Prevalence, incidence and cumulative incidence for the sexual contacts network. (A) Comparison between the individual single source outbreaks (blue, right axes) and the corresponding, averaged prevalence (black, left axes). The infectious period is fixed at *τ* = 91 d. The arrow indicates the maximum averaged prevalence. (B) Mean prevalence *ρ*(*I*_*t*_) (solid curves), incidence *ρ*(*J*_*t*_) (blue bars, right scale) and cumulative incidence *ρ*(*C*_*t*_) (dashed curves). Here, the arrow points at the maximum averaged incidence.

**Fig 4:**
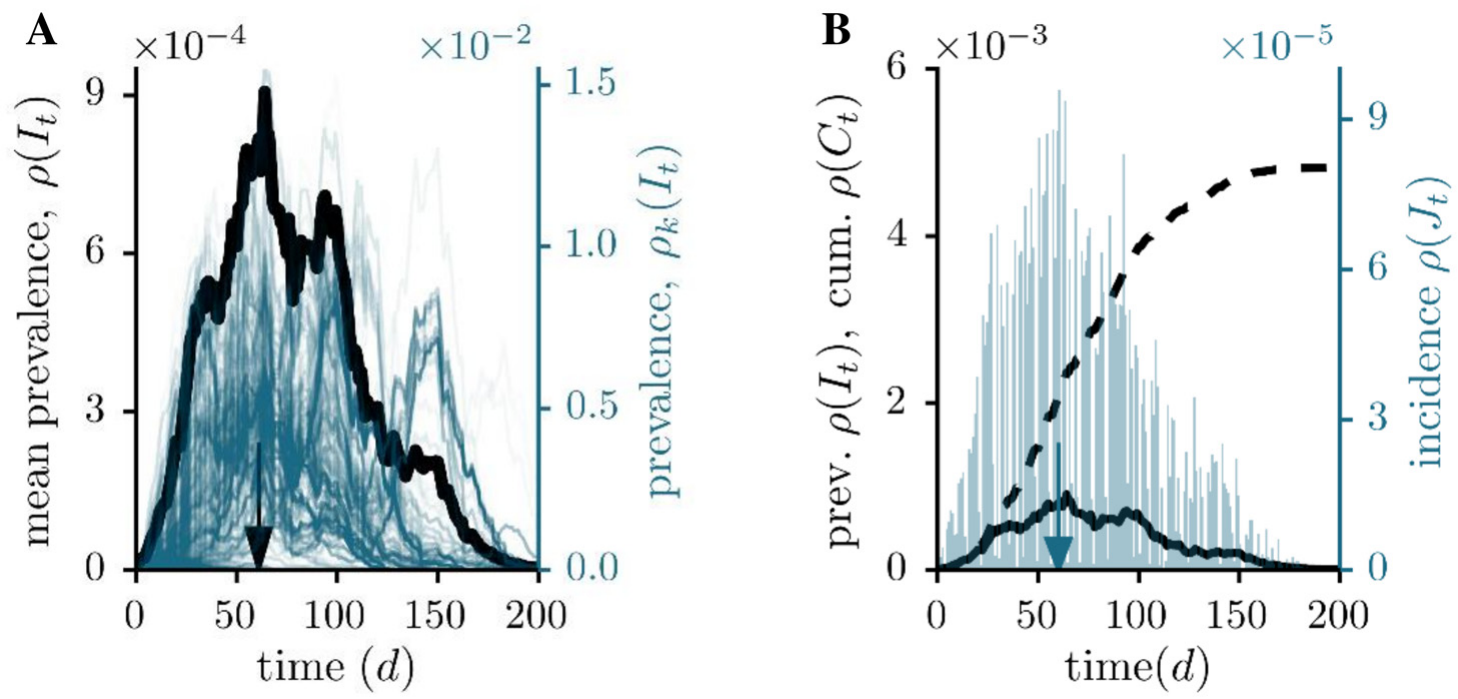
Prevalence, incidence and cumulative incidence for the livestock-trade network. (A) Comparison between the individual single source outbreaks (blue, right axes) and the corresponding, averaged prevalence (black, left axes). The infectious period is fixed at *τ* = 14 d. The arrow indicates the maximum averaged prevalence. (B) Mean prevalence *ρ*(*I*_*t*_) (solid curves), incidence *ρ*(*J*_*t*_) (blue bars, right scale) and cumulative incidence *ρ*(*C*_*t*_) (dashed curves). Here, the arrow points at the maximum averaged incidence.

We list the average fraction of individuals, which have been infected up to the observation time for the temporal networks ($\rho (C_{T}^{\text{temporal}})$) and the corresponding time aggregated graph ($\rho (C_{T}^{\text{static}})$). We use the same recovery times as in Figs 2–4.

In order to compare the result we ran an equivalent experiment on the time aggregated network. We used the same disease parameter and assumed that the infection can spread only to the nearest neighbours within one time step. As a result, every node would be finally infected, independent of the seed of infection (Table 1). The difference to the temporal network amounts to only 17 nodes on average. Therefore, with regard to the total impact of an outbreak a temporal representation of the data might not be necessary.

### Sexual Contacts network

The result is rather different, if we analyse the dynamics on a sexual contacts network from a Brazilian escort website [14] (see Fig 3). 16,730 participants have volunteered to record their sexual interactions online. The data spans the period from September 2002 to October 2008 and given the context of this dataset, allows to study the dynamics of sexually transmitted disease. We follow the numerical analysis of [33] and choose 91 days as the infectious period *τ*. It reflects the most contagious period after an HIV-1 infection, which is followed by a chronical stage with low transmission probability [34]. Furthermore, we ignore the first 1000 days in order to avoid transient effects during the early stage of the growing network [33].

We notice that the mean prevalence peaks after 305 days (Fig 3, panel A), which is followed by a very slow decline. Moreover, we observe in Fig 3B a considerable incidence throughout the observation period. The highest number of new infections is reached after 245 days, which is again a characteristic time that is much longer than the infectious period. Unlike the previous example, the temporal characteristics of the sexual contacts graph lead to a prolonged disease outbreak. This feature is mainly a consequence of the fact that the network is steadily growing [14] and an increasing number of interaction is recorded. The mean cumulative incidence shows that 2.7% of all nodes are affected at the end of the observation time. A comparison with the static network reveals that the total impact would be overestimated by more than one order of magnitude if we used aggregated data (Table 1).

### Livestock-Trade Network

Finally, we apply our formalism to an excerpt of the national German livestock database *HI-Tier* [35], which has been established according to EU legislation. It comprises the movement of pig in a period of 200 days from 2011-01-01 to 2011-07-20. With a time resolution of one day more than 9⋅10^5^ trade transactions (directed links) have been recorded between 70,286 agricultural premises and traders (nodes), respectively. Livestock-trade is considered to be a major transmission route for animal-related diseases like classical swine fever [36]. In a potential outbreak, a detected node would be isolated from the trade network and therefore we can consider the infectious period to be mainly determined by the detection time. In our example shown in Fig 4 we assume a detection time of 14 days.

Similar to the sexual network, we observe a broad evolution of the epidemic with a characteristic time scale of the mean prevalence (64 days) and incidence (60 days), respectively. We note again that both values are much larger than the infectious period, which indicates a slow mixing within the network. At the end of the observation, a fraction of 0.5% (around 340 nodes) would be affected. The corresponding static analysis would yield a result, which is again more than one order of magnitude higher (Table 1).

These findings confirm previous observations [13, 33] that time-respecting paths can lead to a considerable improvement compared to static network analysis. It is of crucial importance to take into account the temporal sequence of the links in contact networks, especially, when the infection dynamics takes place on similar time scales as the network evolution.

### Critical Infectious Period

**Fig 5:**
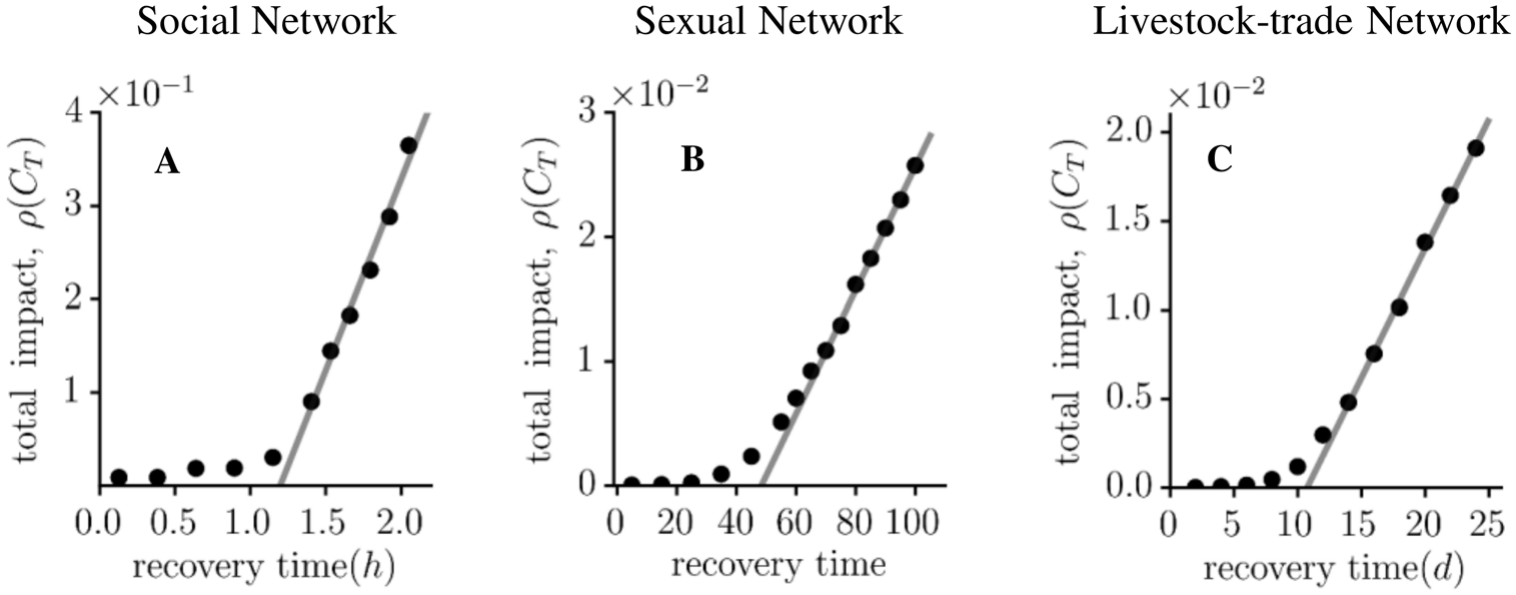
Estimating the critical infectious period for the social (A), the sexual (B) and the livestock-trade network (C). Fraction of nodes, which have been infected up to the observation time as a function of the infectious period *τ*. The grey line is a linear regression through the last 6 data points and the zero crossing gives a rough estimate of the critical infectious period *τ*_*c*_. We found *τ*_*c*_ = 1.20 ± 0.05 hours, *τ*_*c*_ = 48 ± 2 days and *τ*_*c*_ = 10.8 ± 0.3 days for the social, the sexual and the livestock-trade network, respectively. The uncertainties are calculated from the least-squares fit.

In order to further analyse the impact of the infectious period *τ*, we examine the fraction of nodes, which have been infected once during the observation time, i.e. *C*_*T*_. Again, we operate under the assumption that one node is infected at the beginning of the observation. Our formalism given by Eqs (5) and (7b) allows us to calculate efficiently the cumulative incidence *C*_*T*_ at the end of the observation time *T* for every initial condition in dependence on the disease parameter *τ*. This is depicted in Fig 5 for the three considered contact networks.

Focusing on the small-*τ* range, we notice a transition, where the impact of the epidemic changes qualitatively from local to global outbreaks. This behaviour is well known in the literature [6, 23, 37] and indicates the existence of a critical infectious period *τ*_*c*_ or an epidemic threshold under the assumption that the transmission probability is one. It has been shown that the epidemic threshold for SIR infections is related to properties known from percolation theory [29, 30, 38, 39]. Here, a giant connected component emerges if the fraction of links exceeds a certain threshold [40, 41]. We estimate the critical transition point through a linear regression. This approach is motivated by the effective medium theory, which predicts a linear behaviour above the critical value [42]. The result gives an upper bound to the typical transition point. Thus, it can be considered a loose statistical criterion for the point, beyond which a considerable fraction of the network can become infected.

The social network is highly dynamic as observed before and therefore it does not surprise that the critical infectious period is as low as 1.2 hours (Fig 5A). For the sexual contact network, we find that a disease with an infectious period above 48 days can affect a considerable fraction of the network (Fig 5B). Finally, we observe in the pig trade data a critical transition at *τ*_*c*_ = 10.8 days (Fig 5C). This finding may suggest for example that the detection time of any outbreak in the livestock production should be below 11 days in order to minimize the impact of a disease.

Recently, an elegant approach to calculate the epidemic threshold in temporal networks has been proposed [37]. It is based on the mean field assumption that dynamic correlations can be ignored which means that the disease status of a node is independent of its nearest neighbour states. Though unrealistic for some networks [43], this approximation helps to predict the true epidemic threshold to a high degree of accuracy. On the other hand, the matrix-based approach, which we have presented in this paper takes into account all time and topology dependent correlations. It therefore extends existing approaches and allows to deal with networks, where these correlations are crucial.

## Conclusion

We have introduced a formalism to calculate paths for infections in temporal networks, which is based on elementary operations from linear algebra and Boolean arithmetic. In particular, we have extended the concept of unfolding accessibility to include finite infectious periods. We have focused on the susceptible-infected-recovered (SIR) model with a fixed infectious periods *τ* and a transmission probability of unity. Apart from the disease parameter *τ*, the proposed formalism is based entirely on the adjacency matrices of the temporal network, which refer to snapshots of the underlying contact network. Thus, we have been able to deal with the disease dynamics and the temporal network in one unifying framework, taking into account both temporal and topological correlations.

In order to apply our framework to epidemiology, we have derived three accessibility matrices that allow to compute simultaneously the prevalence, incidence and cumulative incidence for different initial conditions. The density of each matrix hence returns the mean value, averaged over all realizations.

From a computational point of view, the time complexity is dominated by the matrix multiplication *A*_*t*_ *I*_*t*_ and the element-wise multiplication *A*_*t*_ *I*_*t*_ ∘ (¬*R*_*t*+1_) in Eq (5a). Both can be implemented efficiently for sparse Boolean matrices. Note however, that the negated term ¬*R*_*t*+1_ becomes dense for a small fraction of recovered nodes. Similarly, we find that well mixing networks, such as the social contacts graph, can lead to a dense prevalence matrix *I*_*t*_ and hence slowdown the performance. Furthermore, we find that the storage complexity grows linearly with the recovery time and quadratically with the number of nodes in the case of a dense accessibility matrix.

We applied the formalism to three real-world networks: conference interactions, sexual contacts, and livestock-trade. In each case, we have presented an analysis based on the average prevalence, incidence, and cumulative incidence and recovered relations between the dynamics of the disease and the features of underlying contact graph. We have also analysed the total fraction of nodes, which have been infected at some point during the observation period depending on the infectious period *τ*. By considering the small-*τ* limit, we have observed a critical value *τ*_*c*_, above which a disease can affect a considerable fraction of the network. This value can be related to the well known basic reproduction number *R*_0_, which has been applied only recently to temporal networks, using an alternative method [37].
